# β-Nd_2_Mo_4_O_15_

**DOI:** 10.1107/S1600536810048609

**Published:** 2010-11-27

**Authors:** Dan Zhao, Fei-Fei Li, Yu-Ming Yao, Chang-An Huan, En-Xiao Zhao

**Affiliations:** a Department of Physics and Chemistry, Henan Polytechnic University, Jiaozuo, Henan 454000, People’s Republic of China

## Abstract

The title compound, dineodymium(III) tetra­molybdate(VI), has been prepared by a flux technique and is the second polymorph of composition Nd_2_Mo_4_O_15_. The crystal structure is isotypic with those of Ce_2_Mo_4_O_15_ and Pr_2_Mo_4_O_15_. It features a three-dimensional network composed of distorted edge- and corner-sharing NdO_7_ polyhedra, NdO_8_ polyhedra, MoO_4_ tetra­hedra and MoO_6_ octa­hedra.

## Related literature

For background to molybdates with rare earth (*RE*) cations, see: Borchardt & Bierstedt (1966 ▶); Ouwerkerk *et al.* (1982 ▶). For the *α*-polymorph of Nd_2_Mo_4_O_15_, see: Naruke & Yamase (2003 ▶). Structures isotypic with β-Nd_2_Mo_4_O_15_ were reported for the Ce (Fallon & Gatehouse, 1982 ▶) and Pr (Efremov *et al.*, 1988*a* ▶) analogues. For the crystal structures, properties and applications of other molybdates with general formula *RE*_2_Mo_4_O_15_, see: *RE* = La (Dubois *et al.*, 2001 ▶); Tb (Naruke & Yamase, 2001 ▶); La, Nd, Sm (Naruke & Yamase, 2003 ▶); Ho (Efremov *et al.*, 1988*b* ▶).

## Experimental

### 

#### Crystal data

Nd_2_Mo_4_O_15_
*M* *_r_* = 912.24
Triclinic, 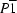

*a* = 7.4000 (6) Å
*b* = 7.4992 (6) Å
*c* = 11.7291 (9) Å
α = 88.916 (2)°
β = 83.957 (1)°
γ = 84.196 (2)°
*V* = 643.94 (9) Å^3^
*Z* = 2
Mo *K*α radiation
μ = 11.77 mm^−1^
*T* = 293 K
0.15 × 0.15 × 0.05 mm

#### Data collection

Bruker SMART 1K CCD diffractometer
Absorption correction: multi-scan (*SADABS*; Bruker, 1997 ▶) *T*_min_ = 0.271, *T*_max_ = 0.591
3620 measured reflections
2390 independent reflections
2268 reflections with *I* > 2σ(*I*)
*R*_int_ = 0.040

#### Refinement

*R*[*F* ^2^ > 2σ(*F* ^2^)] = 0.046
*wR*(*F* ^2^) = 0.128
*S* = 1.05
2390 reflections
191 parameters
Δρ_max_ = 3.38 e Å^−3^
Δρ_min_ = −2.54 e Å^−3^

### 

Data collection: *SMART* (Bruker, 1997 ▶); cell refinement: *SAINT* (Bruker, 1997 ▶); data reduction: *SAINT*; program(s) used to solve structure: *SHELXS97* (Sheldrick, 2008 ▶); program(s) used to refine structure: *SHELXL97* (Sheldrick, 2008 ▶); molecular graphics: *DIAMOND* (Brandenburg, 2004 ▶); software used to prepare material for publication: *SHELXTL* (Sheldrick, 2008 ▶).

## Supplementary Material

Crystal structure: contains datablocks I, global. DOI: 10.1107/S1600536810048609/wm2425sup1.cif

Structure factors: contains datablocks I. DOI: 10.1107/S1600536810048609/wm2425Isup2.hkl

Additional supplementary materials:  crystallographic information; 3D view; checkCIF report

## Figures and Tables

**Fig. 1.**
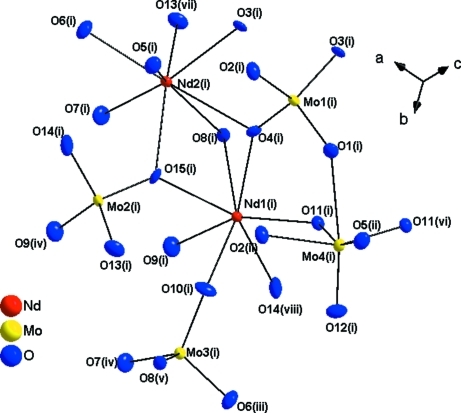
The expanded asymmetric unit of β-Nd2Mo4O15 showing the coordination environments of the Mo and Nd atoms. [Symmetry codes: (i) x, y, z; (ii) 1 - x, 1 - y, 1 - z; (iii) -1 + x, 1 + y, z; (iv) 1 - x, 1 - y, -z; (v) x, 1 + y, z; (vi) -x, 1 - y, 1 - z; (vii) x, -1 + y, z; (viii) -1 + x, y, z.]

**Fig. 2.**
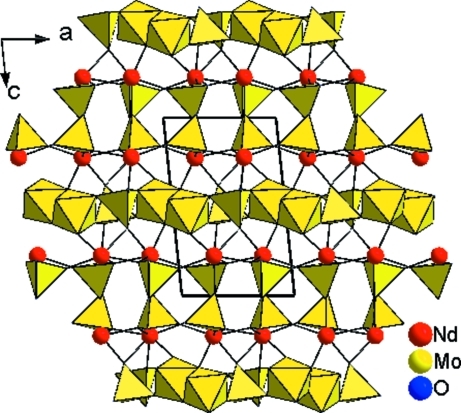
View of the crystal structure of β-Nd2Mo4O15 along [010]. MoO4 and MoO6 units are given in the polyhedral representation.

## supplementary crystallographic information

### Comment

Rare-earth molybdate compounds have been intensively studied due to their diversity and excellent chemical stabilities, as well as their potential applications as laser host phosphors, or as ferroelectric and ferroelastic materials (Borchardt & Bierstedt, 1966; Ouwerkerk *et al.*, 1982). Previous studies of the family of *RE*_2_Mo_4_O_15_ (*RE* is a rare earth metal cation) compounds show that they adopt different structure types, such as monoclinic La_2_Mo_4_O_15_ with *Z* = 4 (Dubois *et al.*, 2001; Naruke & Yamase, 2003); Tb_2_Mo_4_O_15_ (Naruke & Yamase, 2001) and Ho_2_Mo_4_O_15_ (Efremov *et al.*, 1988*b*) with *Z* = 2, or triclinic Nd_2_Mo_4_O_15_ (Naruke & Yamase, 2003) with *Z* = 3. In this paper, we present synthesis and crystal structure of the β-phase of compound Nd_2_Mo_4_O_15_ which is structurally different from the first (*α*-) Nd_2_Mo_4_O_15_ polymorph (Naruke & Yamase, 2003), but is isotypic with Ce_2_Mo_4_O_15_ (Fallon & Gatehouse, 1982) and Pr_2_Mo_4_O_15_ (Efremov *et al.*, 1988*a*) with *Z* = 2.

The structure of β-Nd_2_Mo_4_O_15_ features a three-dimensional framework composed of distorted NdO_7_, NdO_8_, MoO_4_ and MoO_6_ polyhedra, as shown in Fig. 1. There are four crystallographically different Mo atoms in the asymmetric unit. Mo(1), Mo(2), Mo(3) atoms are surrounded by four oxygen atoms within a tetrahedral coordination, while the Mo(4) atom is surrrounded by six oxygen atoms within a considerably distorted octahedral coordination. Two adjacent Mo(4)O_6_ octahedra are connected through edge-sharing, forming Mo_2_O_10_ units. These Mo_2_O_10_ units are interconnected by Mo(1)O_4_ tetrahedra *via* corner-sharing to form an infinite Mo_4_O_14_ chain parallel to  [100]. The distorted environments of the two Nd atoms Nd(1) and Nd(2) are different. While Nd(1) is coordinated by seven oxygen atoms, Nd(2) is coordinated by eight oxygen atoms. The Mo_4_O_14_ chains are linked perpendicularly to the chain direction into a three-dimensional framework *via* isolated Mo(2)O_4_ and Mo(3)O_4_ tetrahedra and by Nd(1)O_7_ and Nd(2)O_8_ polyhedra sharing edges and corners (Fig. 2) .

### Experimental

The finely ground reagents K_2_CO_3_, Nd_2_O_3_, and MoO_3_ were mixed in the molar ratio K: Nd: Mo = 3: 2: 6, were placed in a Pt crucible, and heated at 573 K for 4 h. The mixture was then re-ground and heated at 1273 K for 20 h, then cooled to 673 K at a rate of 3 K h^-1^, and finally quenched to room temperature. A few light-red crystals of the title compound with prismatic shape were obtained.

### Refinement

The highest peak in the difference electron density map equals to 3.38 e/Å^3^ at the distance of 0.92 Å from the Nd(1) site while the deepest hole equals to -2.54 e/Å^3^ at the distance of 1.22 Å from the Nd(2) site.

### Crystal data

**Table:**

Nd_2_Mo_4_O_15_	*Z* = 2
*M**_r_* = 912.24	*F*(000) = 816
Triclinic, *P*1	*D*_x_ = 4.705 Mg m^−^^3^
Hall symbol: -P 1	Mo *K*α radiation, λ = 0.71073 Å
*a* = 7.4000 (6) Å	Cell parameters from 487 reflections
*b* = 7.4992 (6) Å	θ = 2.1–23.0°
*c* = 11.7291 (9) Å	µ = 11.77 mm^−^^1^
α = 88.916 (2)°	*T* = 293 K
β = 83.957 (1)°	Prism, light-red
γ = 84.196 (2)°	0.15 × 0.15 × 0.05 mm
*V* = 643.94 (9) Å^3^	

### Data collection

**Table:**

Bruker SMART 1K CCD diffractometer	2390 independent reflections
Radiation source: fine-focus sealed tube	2268 reflections with *I* > 2σ(*I*)
graphite	*R*_int_ = 0.040
ω scans	θ_max_ = 25.7°, θ_min_ = 1.8°
Absorption correction: multi-scan (*SADABS*; Bruker, 1997)	*h* = −9→8
*T*_min_ = 0.271, *T*_max_ = 0.591	*k* = −9→6
3620 measured reflections	*l* = −14→14

### Refinement

**Table:**

Refinement on *F*^2^	Primary atom site location: structure-invariant direct methods
Least-squares matrix: full	Secondary atom site location: difference Fourier map
*R*[*F*^2^ > 2σ(*F*^2^)] = 0.046	*w* = 1/[σ^2^(*F*_o_^2^) + (0.0986*P*)^2^ + 6.3644*P*] where *P* = (*F*_o_^2^ + 2*F*_c_^2^)/3
*wR*(*F*^2^) = 0.128	(Δ/σ)_max_ < 0.001
*S* = 1.05	Δρ_max_ = 3.38 e Å^−^^3^
2390 reflections	Δρ_min_ = −2.54 e Å^−^^3^
191 parameters	Extinction correction: *SHELXL97* (Sheldrick, 2008), Fc^*^=kFc[1+0.001xFc^2^λ^3^/sin(2θ)]^-1/4^
0 restraints	Extinction coefficient: 0.0080 (7)

### Special details

**Table:**

Geometry. All e.s.d.'s (except the e.s.d. in the dihedral angle between two l.s. planes) are estimated using the full covariance matrix. The cell e.s.d.'s are taken into account individually in the estimation of e.s.d.'s in distances, angles and torsion angles; correlations between e.s.d.'s in cell parameters are only used when they are defined by crystal symmetry. An approximate (isotropic) treatment of cell e.s.d.'s is used for estimating e.s.d.'s involving l.s. planes.
Refinement. Refinement of *F*^2^ against ALL reflections. The weighted *R*-factor *wR* and goodness of fit *S* are based on *F*^2^, conven tional *R*-factors *R* are based on *F*, with *F* set to zero for negative *F*^2^. The threshold expression of *F*^2^ > σ(*F*^2^) is used only for calculating *R*-factors(gt) *etc*. and is not relevant to the choice of reflections for refinement. *R*-factors based on *F*^2^ are statistically about twice as large as those based on *F*, and *R*- factors based on ALL data will be even larger.

### Fractional atomic coordinates and isotropic or equivalent isotropic displacement parameters (Å^2^)

**Table:**

	*x*	*y*	*z*	*U*_iso_*/*U*_eq_	
Nd1	0.24478 (7)	0.41215 (6)	0.22477 (4)	0.0090 (2)	
Nd2	0.67891 (6)	0.09060 (6)	0.22360 (4)	0.0081 (2)	
Mo1	0.43929 (11)	0.25334 (11)	0.52879 (7)	0.0093 (3)	
Mo2	0.72798 (11)	0.57014 (10)	0.12800 (7)	0.0081 (3)	
Mo3	0.22775 (11)	0.92862 (10)	0.12894 (7)	0.0084 (3)	
Mo4	0.09418 (11)	0.67225 (10)	0.52795 (7)	0.0090 (3)	
O11	0.0842 (9)	0.4745 (9)	0.4032 (6)	0.0122 (14)	
O2	0.6680 (10)	0.2917 (10)	0.5608 (6)	0.0180 (15)	
O15	0.5756 (9)	0.4080 (9)	0.1806 (6)	0.0124 (14)	
O8	0.3544 (9)	0.0941 (9)	0.1872 (6)	0.0116 (14)	
O1	0.2954 (10)	0.4305 (9)	0.5914 (6)	0.0161 (15)	
O5	0.8677 (10)	0.2270 (10)	0.3442 (7)	0.0183 (15)	
O7	0.7035 (11)	0.0974 (11)	0.0170 (7)	0.0209 (17)	
O12	−0.0335 (11)	0.8313 (10)	0.4610 (7)	0.0217 (16)	
O4	0.4229 (10)	0.2652 (9)	0.3798 (6)	0.0153 (15)	
O6	0.9983 (10)	0.0078 (11)	0.1526 (7)	0.0231 (17)	
O9	0.2747 (11)	0.4070 (11)	0.0191 (7)	0.0227 (17)	
O10	0.2620 (11)	0.7197 (10)	0.1964 (6)	0.0200 (16)	
O13	0.6638 (11)	0.7841 (10)	0.1853 (7)	0.0207 (16)	
O14	0.9451 (11)	0.4864 (12)	0.1581 (7)	0.0253 (18)	
O3	0.3788 (10)	0.0521 (9)	0.5944 (6)	0.0150 (14)	

### Atomic displacement parameters (Å^2^)

**Table:**

	*U*^11^	*U*^22^	*U*^33^	*U*^12^	*U*^13^	*U*^23^
Nd1	0.0097 (3)	0.0080 (3)	0.0089 (3)	0.0000 (2)	0.0000 (2)	0.0003 (2)
Nd2	0.0096 (3)	0.0058 (3)	0.0091 (3)	−0.0022 (2)	−0.0012 (2)	−0.0003 (2)
Mo1	0.0098 (4)	0.0084 (4)	0.0101 (4)	−0.0026 (3)	−0.0019 (3)	0.0022 (3)
Mo2	0.0086 (4)	0.0075 (4)	0.0084 (4)	−0.0030 (3)	0.0001 (3)	0.0001 (3)
Mo3	0.0089 (4)	0.0080 (4)	0.0088 (4)	−0.0025 (3)	−0.0011 (3)	−0.0016 (3)
Mo4	0.0083 (4)	0.0079 (4)	0.0113 (4)	−0.0026 (3)	−0.0011 (3)	−0.0023 (3)
O11	0.013 (3)	0.013 (3)	0.011 (3)	−0.004 (3)	−0.001 (3)	0.000 (3)
O2	0.017 (4)	0.023 (4)	0.015 (4)	−0.006 (3)	0.000 (3)	0.002 (3)
O15	0.006 (3)	0.011 (3)	0.020 (4)	−0.001 (2)	0.003 (3)	−0.006 (3)
O8	0.010 (3)	0.008 (3)	0.017 (3)	−0.006 (3)	−0.002 (3)	−0.002 (3)
O1	0.019 (4)	0.009 (3)	0.019 (4)	−0.003 (3)	0.000 (3)	−0.001 (3)
O5	0.016 (4)	0.016 (4)	0.024 (4)	−0.004 (3)	−0.001 (3)	−0.004 (3)
O7	0.026 (4)	0.024 (4)	0.012 (4)	−0.001 (3)	0.000 (3)	−0.006 (3)
O12	0.023 (4)	0.016 (4)	0.027 (4)	0.003 (3)	−0.008 (3)	0.000 (3)
O4	0.015 (3)	0.009 (3)	0.019 (4)	0.005 (3)	0.001 (3)	−0.001 (3)
O6	0.012 (4)	0.030 (5)	0.026 (4)	0.002 (3)	0.001 (3)	−0.002 (3)
O9	0.026 (4)	0.027 (4)	0.016 (4)	−0.005 (3)	−0.001 (3)	−0.003 (3)
O10	0.032 (4)	0.015 (4)	0.016 (4)	−0.005 (3)	−0.013 (3)	0.004 (3)
O13	0.031 (4)	0.011 (4)	0.021 (4)	−0.006 (3)	−0.006 (3)	−0.001 (3)
O14	0.015 (4)	0.039 (5)	0.021 (4)	−0.007 (3)	0.003 (3)	0.007 (4)
O3	0.017 (3)	0.008 (3)	0.019 (4)	−0.002 (3)	0.000 (3)	0.008 (3)

### Geometric parameters (Å, °)

**Table:**

Nd1—O11	2.326 (7)	Mo2—O15	1.798 (7)
Nd1—O10	2.339 (7)	Mo3—O6^v^	1.737 (7)
Nd1—O9	2.400 (8)	Mo3—O7^iv^	1.742 (8)
Nd1—O14^i^	2.437 (8)	Mo3—O10	1.749 (7)
Nd1—O15	2.446 (6)	Mo3—O8^vi^	1.814 (6)
Nd1—O8	2.472 (7)	Mo4—O12	1.680 (8)
Nd1—O4	2.528 (7)	Mo4—O5^vii^	1.753 (7)
Nd1—Nd2	3.8158 (7)	Mo4—O11^viii^	1.909 (7)
Nd2—O13^ii^	2.366 (7)	Mo4—O2^vii^	1.989 (7)
Nd2—O3^iii^	2.387 (7)	Mo4—O11	2.115 (7)
Nd2—O5	2.399 (7)	Mo4—O1	2.386 (7)
Nd2—O7	2.411 (8)	Mo4—Mo4^viii^	3.1674 (15)
Nd2—O6	2.444 (7)	O11—Mo4^viii^	1.909 (7)
Nd2—O8	2.480 (7)	O2—Mo4^vii^	1.989 (7)
Nd2—O15	2.484 (7)	O8—Mo3^ii^	1.814 (6)
Nd2—O4	2.742 (7)	O5—Mo4^vii^	1.753 (7)
Mo1—O1	1.739 (7)	O7—Mo3^iv^	1.742 (8)
Mo1—O3	1.758 (7)	O6—Mo3^ix^	1.737 (7)
Mo1—O4	1.764 (7)	O9—Mo2^iv^	1.733 (8)
Mo1—O2	1.823 (7)	O13—Nd2^vi^	2.366 (7)
Mo2—O9^iv^	1.733 (8)	O14—Nd1^x^	2.437 (8)
Mo2—O14	1.734 (8)	O3—Nd2^iii^	2.387 (7)
Mo2—O13	1.753 (7)		
			
O11—Nd1—O10	88.8 (3)	O4—Nd2—Nd1	41.44 (15)
O11—Nd1—O9	153.3 (3)	O1—Mo1—O3	108.8 (3)
O10—Nd1—O9	83.4 (3)	O1—Mo1—O4	107.4 (3)
O11—Nd1—O14^i^	82.8 (3)	O3—Mo1—O4	114.5 (3)
O10—Nd1—O14^i^	82.1 (3)	O1—Mo1—O2	105.5 (3)
O9—Nd1—O14^i^	70.9 (3)	O3—Mo1—O2	109.3 (3)
O11—Nd1—O15	125.3 (2)	O4—Mo1—O2	110.9 (3)
O10—Nd1—O15	81.3 (3)	O9^iv^—Mo2—O14	109.3 (4)
O9—Nd1—O15	78.7 (3)	O9^iv^—Mo2—O13	106.3 (4)
O14^i^—Nd1—O15	146.7 (3)	O14—Mo2—O13	112.1 (4)
O11—Nd1—O8	116.2 (2)	O9^iv^—Mo2—O15	109.1 (4)
O10—Nd1—O8	152.5 (3)	O14—Mo2—O15	107.0 (3)
O9—Nd1—O8	78.6 (3)	O13—Mo2—O15	113.0 (3)
O14^i^—Nd1—O8	110.8 (3)	O6^v^—Mo3—O7^iv^	111.1 (4)
O15—Nd1—O8	75.0 (2)	O6^v^—Mo3—O10	109.0 (4)
O11—Nd1—O4	70.6 (2)	O7^iv^—Mo3—O10	108.4 (4)
O10—Nd1—O4	116.3 (2)	O6^v^—Mo3—O8^vi^	106.6 (4)
O9—Nd1—O4	135.5 (2)	O7^iv^—Mo3—O8^vi^	109.7 (3)
O14^i^—Nd1—O4	146.5 (2)	O10—Mo3—O8^vi^	112.0 (3)
O15—Nd1—O4	66.7 (2)	O12—Mo4—O5^vii^	104.3 (4)
O8—Nd1—O4	66.3 (2)	O12—Mo4—O11^viii^	102.4 (4)
O11—Nd1—Nd2	116.20 (17)	O5^vii^—Mo4—O11^viii^	95.7 (3)
O10—Nd1—Nd2	120.5 (2)	O12—Mo4—O2^vii^	97.0 (4)
O9—Nd1—Nd2	89.6 (2)	O5^vii^—Mo4—O2^vii^	97.9 (3)
O14^i^—Nd1—Nd2	148.9 (2)	O11^viii^—Mo4—O2^vii^	152.7 (3)
O15—Nd1—Nd2	39.66 (16)	O12—Mo4—O11	94.5 (3)
O8—Nd1—Nd2	39.68 (15)	O5^vii^—Mo4—O11	160.8 (3)
O4—Nd1—Nd2	45.87 (15)	O11^viii^—Mo4—O11	76.3 (3)
O13^ii^—Nd2—O3^iii^	73.9 (3)	O2^vii^—Mo4—O11	83.4 (3)
O13^ii^—Nd2—O5	129.8 (3)	O12—Mo4—O1	170.4 (3)
O3^iii^—Nd2—O5	75.9 (3)	O5^vii^—Mo4—O1	83.9 (3)
O13^ii^—Nd2—O7	79.6 (3)	O11^viii^—Mo4—O1	81.3 (3)
O3^iii^—Nd2—O7	153.0 (3)	O2^vii^—Mo4—O1	76.7 (3)
O5—Nd2—O7	126.9 (3)	O11—Mo4—O1	77.7 (3)
O13^ii^—Nd2—O6	80.7 (3)	O12—Mo4—Mo4^viii^	100.5 (3)
O3^iii^—Nd2—O6	107.9 (3)	O5^vii^—Mo4—Mo4^viii^	133.6 (3)
O5—Nd2—O6	71.7 (3)	O11^viii^—Mo4—Mo4^viii^	40.4 (2)
O7—Nd2—O6	72.0 (3)	O2^vii^—Mo4—Mo4^viii^	117.3 (2)
O13^ii^—Nd2—O8	79.2 (3)	O11—Mo4—Mo4^viii^	35.83 (18)
O3^iii^—Nd2—O8	91.6 (2)	O1—Mo4—Mo4^viii^	76.52 (17)
O5—Nd2—O8	140.7 (2)	Mo4^viii^—O11—Mo4	103.7 (3)
O7—Nd2—O8	78.5 (3)	Mo4^viii^—O11—Nd1	122.5 (3)
O6—Nd2—O8	146.8 (3)	Mo4—O11—Nd1	133.7 (3)
O13^ii^—Nd2—O15	147.6 (2)	Mo1—O2—Mo4^vii^	136.8 (4)
O3^iii^—Nd2—O15	124.5 (2)	Mo2—O15—Nd1	135.0 (4)
O5—Nd2—O15	82.5 (2)	Mo2—O15—Nd2	123.6 (3)
O7—Nd2—O15	77.3 (3)	Nd1—O15—Nd2	101.4 (2)
O6—Nd2—O15	112.6 (3)	Mo3^ii^—O8—Nd1	126.2 (3)
O8—Nd2—O15	74.2 (2)	Mo3^ii^—O8—Nd2	132.4 (3)
O13^ii^—Nd2—O4	119.7 (3)	Nd1—O8—Nd2	100.8 (2)
O3^iii^—Nd2—O4	63.0 (2)	Mo1—O1—Mo4	136.9 (4)
O5—Nd2—O4	78.3 (2)	Mo4^vii^—O5—Nd2	152.6 (4)
O7—Nd2—O4	129.9 (2)	Mo3^iv^—O7—Nd2	165.9 (5)
O6—Nd2—O4	150.0 (2)	Mo1—O4—Nd1	144.6 (4)
O8—Nd2—O4	63.0 (2)	Mo1—O4—Nd2	122.6 (3)
O15—Nd2—O4	62.9 (2)	Nd1—O4—Nd2	92.7 (2)
O13^ii^—Nd2—Nd1	118.7 (2)	Mo3^ix^—O6—Nd2	168.5 (5)
O3^iii^—Nd2—Nd1	99.86 (17)	Mo2^iv^—O9—Nd1	171.6 (5)
O5—Nd2—Nd1	105.23 (18)	Mo3—O10—Nd1	156.8 (4)
O7—Nd2—Nd1	88.49 (19)	Mo2—O13—Nd2^vi^	159.0 (5)
O6—Nd2—Nd1	150.0 (2)	Mo2—O14—Nd1^x^	169.8 (5)
O8—Nd2—Nd1	39.53 (15)	Mo1—O3—Nd2^iii^	143.0 (4)
O15—Nd2—Nd1	38.92 (15)		

Symmetry codes: (i) *x*−1, *y*, *z*; (ii) *x*, *y*−1, *z*; (iii) −*x*+1, −*y*, −*z*+1; (iv) −*x*+1, −*y*+1, −*z*; (v) *x*−1, *y*+1, *z*; (vi) *x*, *y*+1, *z*; (vii) −*x*+1, −*y*+1, −*z*+1; (viii) −*x*, −*y*+1, −*z*+1; (ix) *x*+1, *y*−1, *z*; (x) *x*+1, *y*, *z*.

## References

[bb1] Borchardt, H. J. & Bierstedt, P. E. (1966). *Appl. Phys. Lett.* **8**, 50–52.

[bb2] Brandenburg, K. (2004). *DIAMOND* Crystal Impact GbR, Bonn, Germany.

[bb3] Bruker (1997). *SMART*, *SAINT* and *SADABS* Bruker AXS Inc., Madison, Wisconsin, USA.

[bb4] Dubois, F., Goutenoire, F., Laligant, Y., Suard, E. & Lacorre, P. (2001). *J. Solid State Chem.* **159**, 228–233.

[bb5] Efremov, V. A., Davydova, N. N., Gokhman, L. Z., Evdokimov, A. A. & Trunov, V. K. (1988*b*). *Zh. Neorg. Khim.* **33**, 3005–3010.

[bb6] Efremov, V. A., Davydova, N. N. & Trunov, V. K. (1988*a*). *Zh. Neorg. Khim.* **33**, 3001–3004.

[bb7] Fallon, G. D. & Gatehouse, B. M. (1982). *J. Solid State Chem.* **44**, 156–161.

[bb8] Naruke, H. & Yamase, T. (2001). *Acta Cryst.* E**57**, i106–i108.

[bb9] Naruke, H. & Yamase, T. (2003). *J. Solid State Chem.* **173**, 407–417.

[bb10] Ouwerkerk, M., Kellendonk, F. & Blasse, G. (1982). *J. Chem. Soc. Faraday Trans.* **2**, 603–611.

[bb11] Sheldrick, G. M. (2008). *Acta Cryst.* A**64**, 112–122. 10.1107/S0108767307043930 18156677

